# Central obesity and its associated factors among cancer patients at the University of Gondar Comprehensive Specialized Hospital, Northwest Ethiopia

**DOI:** 10.3389/fonc.2023.1150303

**Published:** 2023-04-12

**Authors:** Meseret Derbew Molla, Haileab Fekadu Wolde, Ephrem Tafesse Teferi, Anteneh Ayelign Kibret

**Affiliations:** ^1^ Department of Biochemistry, School of Medicine, College of Medicine and Health Sciences, University of Gondar, Gondar, Ethiopia; ^2^ Department of Epidemiology and Biostatistics, Institute of Public Health, College of Medicine and Health Sciences, University of Gondar, Gondar, Ethiopia; ^3^ Department of Internal Medicine, School of Medicine, College of Medicine and Health Sciences, University of Gondar, Gondar, Ethiopia; ^4^ Department of Human Anatomy, School of Medicine, College of Medicine and Health Sciences, University of Gondar, Gondar, Ethiopia

**Keywords:** cancer, central obesity, body mass index, associated factors, behavioral factors

## Abstract

**Purpose:**

Obesity, especially the hidden type of obesity (central obesity), has been believed to be the major risk factor for developing and progressing non-communicable diseases, including cancers. However, there are limited studies regarding the issue in Ethiopia and the study area. Therefore, this study aimed to evaluate the magnitude of central obesity and its associated factors among cancer patients visited the oncology unit of the University of Gondar Comprehensive Specialized Hospital.

**Methods:**

An institutional-based cross-sectional study was conducted from January 10 to March 10, 2021. A total of 384 study participants were enrolled using a systematic sampling technique. The data were collected using a semi-structured interviewer-administered questionnaire and were pretested to address the quality of assurance. The weight of the participants was assessed using body mass index (BMI) and central obesity. Both bivariate and multivariate logistic regressions were conducted to identify the factors associated with central obesity, and p-values less than 0.05 with multivariate were considered statistically significant associations.

**Result:**

Most respondents (60.16%) were stage I cancer patients. The study found that about 19.27% of the participants were prevalent central obesity, and none of them were obese by body mass index (BMI) categorization criteria. However, about 12.24% and 7.03% of the participants were found to be underweight and overweight, respectively. The variables associated with central obesity were sex (AOR=14.40; 95% CI: 5.26 - 39.50), occupation (AOR=4.32; 95%CI: 1.10 - 17.01), and residency (AOR=0.30; 95% CI: 0.13 - 0.70).

**Conclusion:**

A significant number of the respondents (19.27%) were centrally obese. Being female, urban residency and having an occupation other than a farmer, merchant, and governmental were the factors associated with central obesity. Hence, cancer patients may be centrally obese with average body weight.

## Background

Non-communicable diseases have become major public health problems worldwide (1). They are the leading cause of death in both developed and under-developing countries. Of these, cancers are the second most common prevalent disease next to cardiovascular diseases (CVDs) (2). Globally, around 18.1 million of the population is affected by cancer problems. Besides, about 9.6 million people are estimated to die yearly from cancer and related complications (3). The problem is becoming rapidly prevalent in low and middle-income countries (LMICs), especially in sub-Saharan African countries, and it has been estimated that in 2050, the majority (70%) of annual cancer case incidence will be located in LMICs (4). The absence of screening and failure to diagnose at an early stage of cancer cases makes it difficult to overcome the burden of cancer problems in LMICs. Moreover, treatment is usually compromised fordifferent reasons, such as limied of skilled manpower, facilities, and economic restriction (5). Therefore, screening for early identification of cancer cases and associated factors are believed to be an ideal way to limit the progression and development of adversative outcomes.

Substance abuse like cigarette smoking, alcohol drinking and chat chewing, frequent utilization of processed food, being overweight or obese, and being older are the most common factors associated with the development and proical characteristics of the study participants among adults gression of most cancers (6). Although the association between obesity and cancer development is still controversial, several reports have approved a strong positive association. For instance, studies done across the world showed that obesity has been directly associated with the occurrence of breast cancer (7), ovarian cancer (8), liver cancer (9), endometrial cancer (10), esophageal adenocarcinoma (11), kidney cancer (12), colorectal cancer, gallbladder cancer (13) and thyroid cancer (14). In particular, the hidden type of obesity (central or visceral obesity) is highly dangerous and is usually associated with the development of several NCDs, including cancers, than that of general obesity (identified by BMI) (15, 16). Currently, dietary modifications, controlling the accumulation of fat around the abdominal wall, weight control, and frequent exercise are recommended as a supplementary therapeutic option to limit the progression of cancer cases (17, 18). Globally, the magnitude of abnormal or excessive fat accumulation which is measured by BMI and/or central obesity among cancer patients, is becoming a remarkable problem in the last decades (19). Although the attribution of obesity for cancer incidence varies among each cancer type, it is estimated to reach up to 44% for esophageal adenocarcinoma and 54% for bladder cancer development (20). However, to the best of our search, there are limited studies regarding the issue in Ethiopia and the study area. Besides, the burdens of central obesity have not got equal attention as general obesity among cancer patients worldwide, and this gives great value to the novelty of our study. Consequently, central obesity is becoming one of the major public health problems for the general population of Ethiopia (21–25). As our study is the first among Ethiopian cancer patients that focused on the magnitude of central obesity, the findings will be filled the knowledge gap on the burden of central obesity for Ethiopian cancer patients and can act as a baseline for further researchers with large sample sizes and multi-centered study area towards the impact of central obesity among Ethiopian cancer patients. The finding of this study will also give valuable input for clinicians to give attainable attention to general and central obesity aimed at cancer patients. Hence, we aimed to evaluate the magnitude of central obesity and its associated factors among cancer patients visiting the Oncology ward of the University of Gondar Comprehensive Specialized Hospital.

## Methods

### Study design and setting

An institution-based cross-sectional study was conducted among cancer patients from January to March 2021. It was accompanied by the University of Gondar Comprehensive Specialized Hospital (UoGCSH), Oncology ward. During the data collection period, the oncology ward has ten beds, serving more than 100 cancer patients annually.

### Participants and sample size

All adult cancer patients who were ≥18 years coming to the Oncology ward were taken as the study’s source population. Of these, who were in the Oncology ward during the study period were recruited as study participants. Severely ill patients who were unable to communicate and did not have attendant, clinically confirmed pregnant women, and edematous and abdominal distension patients were excluded from the study. To determine the sample size of the study, we used a single population formula using a 50% expected proportion of central obesity, 95% confidence level, and a 5% margin of error. By considering the 5% non-response rate, the final size was computed to be 403. A systematic sampling technique with a skip interval of two was implemented to select the sample of the participants.

### Data collection procedure

The data were collected using a semi-structured interviewer-administered questionnaire. It was prepared using related pieces of literature in an international language (English), then translated into the Ethiopian national language (Amharic), and then re-translated back to English to check the consistency. The questionnaire includes the general characteristics of the participants, substance abuse like smoking, alcohol drinking, and Khat chewing, and the factors that are believed to be associated with the independent outcome. The data were collected by trained Health Professionals such as Nurses and Public Health Professionals under the supervision of the principal investigator and oncologist. The completeness and consistency of the data were checked daily by the principal investigator. The substance abuse habits of the participants were assessed using a dichotomous yes and no questionnaire with expanded questions for those who responded “yes” about the amount, duration, and frequency of alcohol drinking, khat chewing, and cigarette smoking habits. Thus, alcohol drinkers were defined as any alcoholic product, including locally prepared alcoholic beverages intake at least twice per week for the last year regardless of the amount, otherwise defined as non-drinkers (26). Furthermore, those individuals who have smoked cigarettes for the last year were defined as smokers unless defined as non-smokers. Khat chewers were defined similarly to smokers (27). The physical activity of the participants was grouped based on the World Health Organization and international physical activities analysis guidelines as vigorous, moderate, and poor physical activities. Any activity that causes a substantial increase in breathing or heart rate (e.g., running, carrying, or lifting heavy loads, digging, or construction work) that continued for at least 30 min for a minimum of three days per week was defined as vigorous physical activity. Besides, any activity that causes a small increase in breathing or heart rate (brisk walking or carrying light loads) that continued for at least 30 min for at least three days per week or five or more days of these activities for at least 20 min per day or ≥3 days of vigorous-intensity activity per week of at least 20 min per day was defined as moderate physical activity. The respondents who did not fulfill both vigorous and moderate intensity activity were grouped under poor physical activators (28).

### Physical measurements

Anthropometric (physical) measurements such as weight, height, waist circumference, and blood pressure were measured through adjusted equipment. The weight and height of the participants were measured in kilograms and centimeters in barefoot, respectively to calculate body mass index (BMI). Consequently, BMI was grouped into underweight (BMI ≤18.5 kg/m^2^), normal weight (BMI between 18.5 and 24.9 kg/m^2^), overweight (BMI between 24.9 and 30 kg/m^2^), and obesity (BMI≥30 kg/m^2^) (29). The Waist circumference was measured to define independent outcome (central obesity). Waist circumference was measured in centimeters at the narrowest mid-point between the lower margin of the lowest palpable rib and the top of the iliac crest with flexible plastic tape without heavy outdoor closing. Then, the participants with a waist circumference of >94 cm for males and >80 cm for females were defined as centrally obese (30). The Blood pressure (BP) of the respondents was measured three times with at least a five-minute interval in each measurement in a sitting position using a standardized mercury sphygmomanometer with an appropriate cuff size that covers two-thirds of the upper arm. It was measured with at least five minutes or 30 minutes rest for those who take hot drinks like coffee on their left arm. Then, the average BP measurement was taken, and elevated BP (hypertension) was defined as if systolic blood pressure (SBP) is ≥140mg/dl or diastolic blood pressure (DBP) is ≥90mg/dl or current use of the anti-hypertensive drugs (31).

### Data processing and analysis

The data was entered into Epidata version 3.1 to check the completeness and analyzed using STATA 14 software. The participants’ socio-demographic, behavioral, and clinical characteristics were described through descriptive statistics and presented using tables and narration. The factors associated with the independent variable were identified using the binary logistic regression model. The multivariable logistic regression was implemented to detect the Adjusted Odds Ratio (AOR). The 95% confidence interval was estimated to show the strength of the associations. Hence, a p-value of less than 0.05 in the multivariable logistic regression analysis was used to declare the statistically significant association of the independent variables with central obesity. Hosmer and Lemeshow’s goodness of fit test was used to check the goodness of fit of the model.

## Results

### Socio-demographic characteristics of the participants

A total of 384 study participants with a 95.3% response rate were completed and enrolled in the analysis. Of these, most of the participants were under the age range of 41-60 years (55.99%) and females (51.30%). Most of the participants were coming from a rural area (64.4%) and more than two-thirds of the participants were Orthodox Christianity followers (63.3%). Regarding bad behavioral practices, about 11.7%, 46.6%, and 26.8% of the study participants were current smokers, alcohol drinkers, and khat chewers, respectively. Out of the total participants, only 13.02% of them had vigorous physical activity (**Table 1**).

**Table 1:** Socio-demographic characteristics of the study participants among adults in urban areas of Northwest Ethiopia, 2022 (n=384).

Characteristics	Number	Percent
Age (years)		
Mean ± SD	49.34 ± 0.66	
Age group		
18-40	99	25.78
41-60	215	55.99
>60	70	18.23
Sex		
Male	187	48.70
Female	197	51.30
Religion		
Orthodox	243	63.28
Muslim	91	23.70
Protestant	45	11.72
Others	5	1.30
Occupation		
Farmer	144	37.50
Merchant	82	21.35
Governmental Others	119 39	30.99 10.16
Wealth index		
Poor	112	29.17
Medium	248	64.58
Rich	24	6.25
Current smoking status		
Yes	45	11.72
No	339	88.28
Alcohol drinking status		
Yes	179	46.61
No	205	53.39
Khat chewing		
Yes	103	26.82
No	281	73.18
Physical activity status		
Vigorous	50	13.02
Moderate	132	34.38
Poor	202	52.60
Type of dietary oil		
Solid	212	54.38
Liquid	172	45.62

Other occupation includes students, stay-at-home spouses, unemployed and retired workers.

### Clinical characteristics of the study participants

About 4.17%, 14.32%, 8.59%, 6.77%, 15.63%, 15.10%, 3.39%, and 32.03% of the participants were diagnosed with lung, breast, cervical, ovarian, hematologic, gastrointestinal, skin, and another type of cancers, respectively. Near to two-thirds of the participants were diagnosed with stage I cancer (60.16%) and 9.11% of them were under an advanced stage or stage IV. The majority of study participants (46.4%) were diagnosed with a duration of three to seven months, and more than two-thirds of the participants (68.8%) were not characterized by cancer metastasis. Most of the participants (82.6%) were already under treatment; of these about 25.24%, 38.8%, and 35.96% were treated with chemotherapy, surgery, and combined chemotherapy & surgery, respectively. Moreover, 36.2% and 16.67% of the study participants had hypertension and diabetes comorbidities, respectively (**Table 2**).

**Table 2:** Clinical characteristics of the study participants among adults in urban areas of Northwest Ethiopia, 2022 (n=384).

Variable	Frequency	%
Type of cancer		
Lung cancer	16	4.17
Breast cancer	55	14.32
Cervical cancer	33	8.59
Ovarian cancer	26	6.77
Hematologic cancer	60	15.63
Gastrointestinal cancer	58	15.10
Skin cancer	13	3.39
Others	123	32.03
Stage of cancer		
Stage I	231	60.16
Stage II	68	17.71
Stage III	50	13.02
Stage IV	35	9.11
Duration of cancer diagnosed (in months)		
≤2	122	31.77
3-7	178	46.35
≥8	84	21.88
Metastasis		
Yes	120	31.25
No	264	68.75
Cancer pain		
Yes	230	59.90
No	154	40.10
Treatment for cancer		
Yes	317	82.55
No	67	17.45
Type of treatment		
Chemotherapy	80	25.24
Surgery	123	38.80
Combined Chemotherapy & Surgery	114	35.96
Duration of cancer treatment		
≤2	86	27.13
3-6	157	49.53
≥7	74	23.34
Hypertension		
Yes	139	36.20
No	245	63.80
Hyperglycemia		
Yes	64	16.67
No	320	83.33

### Determination of central obesity using waist circumference definition criteria and weight status of the participants

This study found that 19.27% (95% CI; 15.61- 23.54) of the cancer patients were centrally obese; of these, the majority (91.89%) were females. However, only 7.03% (95% CI; 4.86 - 10.07) of the study participants were overweight and none of them were obese based on the BMI categorization criteria. Besides, about 12.24% (95% CI; 9.31 -15.93) of the study participants were found to be underweight (**Table 3**).

**Table 3:** Prevalence of central obesity and weight status of the cancer patients at the University of Gondar Specialized Comprehensive Hospital, Northwest Ethiopia, 2022 (n=384).

	Male (%)	Female (%)	Total	Prevalence (95%CI)
Central obesity using waist circumstance				
Centrally obese	6 (8.11)	68 (91.89)	74	19.27 (15.61- 23.54)
Not centrally obese	181 (58.39)	129 (41.61)	310	80.73 (76.45 - 84.38)
Weight Status				
Underweight	22 (11.76)	25 (12.69)	47	12.24 (9.31 -15.93)
Normal	156 (83.42)	154 (78.17)	310	80.73 (76.45 - 84.39)
Overweight	9 (4.81)	18 (9.14)	27	7.03 (4.86 - 10.07)

CI, confidence interval.

### The distribution of central obesity across the type of cancer patients

**Figure 1:**
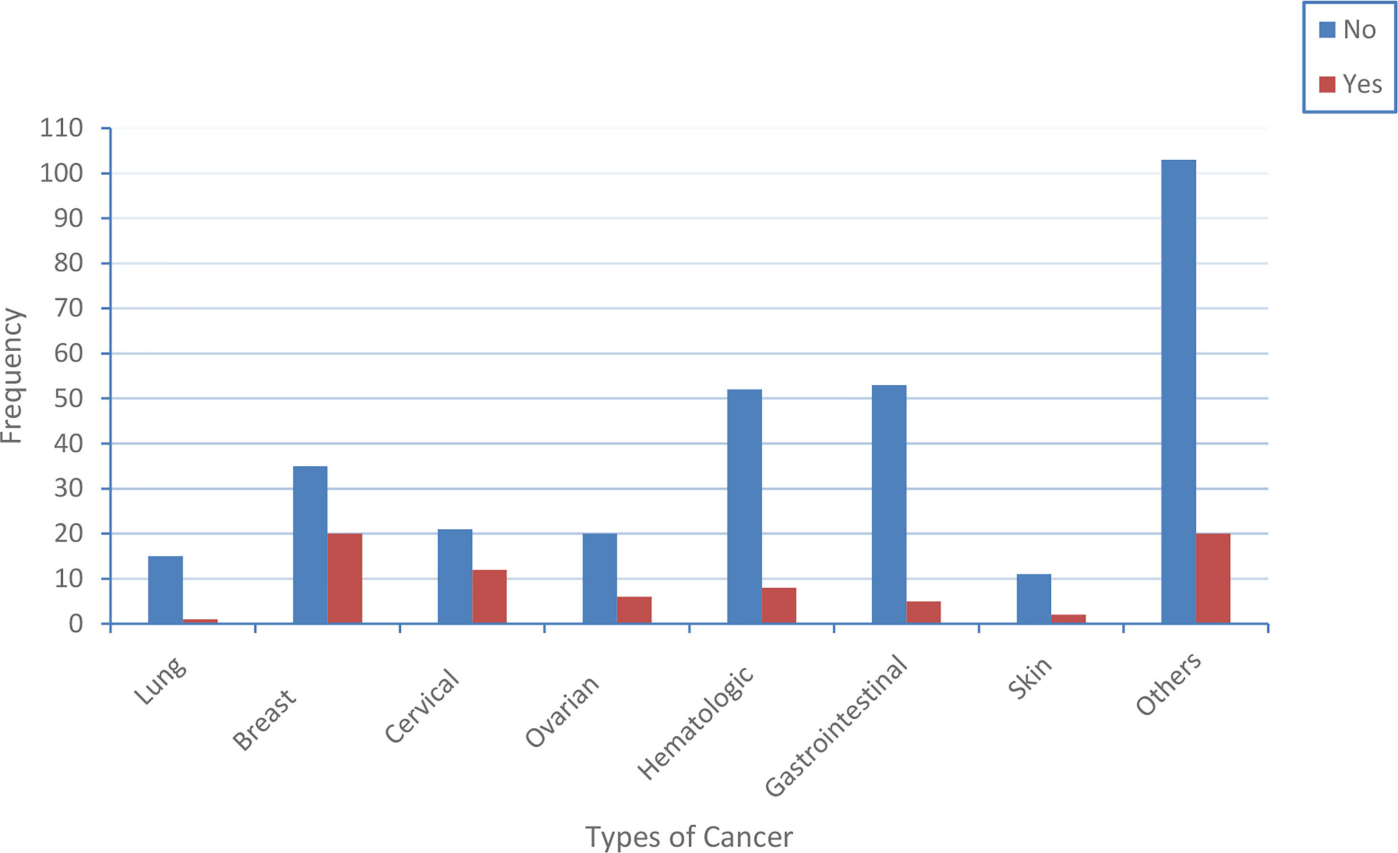
The distribution of central obesity across the type of cancer patients. The blue and red color indicates the proportion of cancer patients that have central obesity and do not have central obesity, respectively.

This study has shown the distribution of central obesity across cancer patients. Accordingly, we found that about 1 out of 15 lung cancer patients, 20 out of 35 breast cancer patients, 12 out of cervical cancer patients, 6 out of 20 0varian cancer patients, 8 out of 52 hematologic cancer patients, 5 out of 53 gastrointestinal cancer patients, 2 out of skin cancer patients, and 20 out of 103 other types of cancer patients were centrally obese (**Figure 1**).

### Factors associated with central obesity among study participants

The study analyzed the multinomial logistic analysis for central obesity to determine its association with the sociodemographic, behavioral, and clinical characteristics of cancer patients. Thus, being female (p<0.001), having urban residency (p=0.002), and having occupations other than a farmer, merchant, and governmental (p=0.036) were found to be the factors associated with central obesity. The probability of having central obesity was 14 times higher among female participants compared to that of male cancer participants (AOR=14.40; 95 CI: 5.26 - 39.50). The odds of having central obesity were 4 times higher among study participants with an occupation other than a farmer, merchant, and governmental as compared to that of the farmers (AOR=4.32; 95%CI: 1.10 - 17.01). Urban residents were less likely to have central obesity by 30% compared to rural residents (AOR=0.30; 95CI: 0.13 - 0.70) (**Table 4**).

**Table 4:** The multivariable logistic regression analysis that identified the factors associated with central obesity among cancer patients at the University of Gondar Specialized Comprehensive Hospital, Northwest Ethiopia, 2022 (n=384) .

Characteristics	Central obesity			
	Yes	No	AOR (95%CI)	P-value
Age category (years)				
<50	36	160	1	
≥50	38	150	1.25 (0.63 - 2.50)	0.517
Sex				
Male	6	181	1	
Female	68	129	14.40 (5.26 - 39.50)	0.000**
Residency				
Rural	61	185	1	
Urban	12	124	0.30 (0.13 - 0.70)	0.002
Occupation				
Farmer	19	125	1	
Merchant	23	59	1.51 (0.54 - 4.20)	0.428
Governmental	23	93	1.81 (0.70 - 4.65)	0.220
Others	6	33	4.32 (1.10 - 17.01)	0.036
Wealth index				
Poor	14	98 191	1	
Medium	57	21	1.55 (0.68 -3.52)	0.290
Rich	3		1.45 (0.28 - 7.47)	0.654
Type of dietary oil				
Liquid	35	137	1	
Solid	39	166	0.60 (0.29 - 1.24)	0.174
Alcohol drinking status				
No	54	129	1	
Yes	20	181	0.39 (0.20 - 0.81)	0.105
Smoking status				
No	67	255	1	
Yes	7	55	2.12 (0.60- 7.55)	0.243
Khat chewing status				
No	17	86 224	1	
Yes	57		1.00 (0.41 - 2.44)	0.998
Physical activity status				
Vigorous	7	43 105	1	
Moderate	27	162	1.15 (0.35 - 3.84)	0.811
Poor	2740		0.95 (0.31 - 2.96)	0.936
Type of cancer				
Lung	15	1	1	
Breast	35	20	0.107 (0.0031- 3.68	0.216
Cervical	21	12	0.102 (0.0027 - 3.81)	0.216
Ovarian	20	6	0.039 (.001 - 1.55)	0.085
Hematologic	52	8	0.370 (0.02 - 5.94)	0.483
Gastrointestinal	53	5	0.058 (0.02- 2.10)	0.120
Skin	11	2	0.267 (0.01 - 14.15)	0.514
Others	103	20	0.170 (0.01- 5.10)	0.308
Duration since diagnosis				
<4 month	37	196	1	
≥4 month	37	114	1.16 (0.52 - 2.59)	0.713
Treatment Type				
Chemotherapy	10	70	1	
Surgery	25	98	1.03 (0.37 - 2.8)	0.957
Combined therapy	28	86	1.08 (0.39 -2.95)	0.877
Duration since treatment				
<3 month or no	19	103	1	
3-6 month	36	142	0.90 (0.362.21)	0.818
>6 month	19	65	1.45 (0.46 - 4.56)	0.518

1 is an indication of reference point, ** is an indication of statistically significant at p-value < 0.001, AOR, adjusted odds ratio; CI, confidence interval.

## Discussion

This is the first study that revealed the most dangerous type of obesity or hidden type of obesity (central obesity) and its association among cancer patients in Ethiopia and the study area.

The study found that a significant proportion of study participants (19.27%) were centrally obese among cancer patients that follow at UOGSCH. However, none of the study participants were found to be obese according to the BMI definition criteria of obesity. Even the prevalence of overweight (7.03%) was lower than compared to that of central obesity, and about 12.24% of the participants were confirmed to be underweight. Most researchers are advised to check fat accumulation around the abdominal wall, which is usually identified through waist circumference measurement, than that of general or peripheral obesity (via BMI) to know the exact pathogenic cause of obesity for the initiation and progression of inflammatory associated chronic diseases, including cancers (32–34). With normal or low BMI levels, people may develop central obesity since BMI can be affected by muscular mass (16, 35). Thus, our finding also revealed that the high burden of central obesity was encountered when compared to that of general obesity among the study participants. Abdominal/visceral obesity (identified by WC) is usually associated with metabolic changes which could affect the pathogenesis of tumor cells. However, the association between obesity and cancers is controversial yet. Some study finding proved their strong positive association, and some others have found no association. However, it has been evidenced that weight gain or obesity is responsible for around 20% of all cancer cases (20). Moreover, several studies and review articles have publicized that over-accumulation of fat around the abdominal wall is the main cause of inflammation cascade initiation, which is one of the most common problems encountered in cancer pathogenesis (36–38). In an individual with visceral obesity, adiposity can be directly encapsulated for tumor development through response to the obesity which is usually associated with the direct release of pro-inflammatory cytokines, hormones (peptide and steroid hormones), and growth factors (39).

The prevalence of central obesity in this study is extremely lower than that of a study done by L.A. Healy et al. (40). This may be due to the study population variation since the current study includes all cancer patients attending the hospital regardless of the metabolic syndrome categorization, whereas the L.A. Healy et al. initially categorizes the study participants as metabolic and non-metabolic participants. As central obesity is one of the major components of metabolic syndrome, the prevalence can be significantly elevated in their study finding. The finding of central obesity was also lower than a study conducted in Africa (41). The discrepancy may be due to the restriction of the study population. The study done among the African population was focused on male cancer patients affected by prostate cancer. The study design may also be different from the current study. Several studies have also been conducted on the association of having central obesity and the progression of tumors (40–42). Although the prevalence of central obesity is a double-digit in the current study without being obese based on BMI classification criteria, it is much lower than studies done in Ethiopia among the general population (21–25). These discrepancies are due to the variation of study participants between studies. Our study focused on cancer patients with disease or medication associated with weight loss. Most studies done about abdominal obesity and cancer targeting their association which makes difficult for further comparison of our finding with other similar studies. This finding will bring for the investigation of further studies and attention for central obesity among cancer patients.

In the current study, the probability of having central obesity was 14 times higher among female participants compared to its counterpart. Although the exact molecular link between being female and central obesity development is not exactly clear, different studies have reported similar findings, even among non-cancer study participants (21, 43, 44). This might be justified as females are naturally fattier than that of males, which may contribute to the re-distribution of fat accumulation towards the visceral area. Consequently, the accumulation of this fat will end up with central obesity. Another possible reason may be due to females may have a sedentary lifestyle and less access to different leaflets that showed the precautions of central obesity for health (45). Most of the population living in developing countries; including Ethiopia do not have enough awareness about central obesity as they estimate their weight using BMI. However, central obesity found among normal BMI groups is the most dangerous as mentioned earlier. It is not also surprising that females living in developing countries have less access to work outside the home, making it more difficult to get different leaflets. Besides, most females have focused on technical activities rather than labor work, and central obesity is frequently associated with those individuals who didn’t undergo physical exercise or labor work (45–47).

The study also revealed that occupation was statistically associated with central obesity among cancer patients. The odds of having central obesity were 4.3 times higher among study participants with occupations other than a farmer, merchant, and governmental than the farmers. We used farmers as reference groups because farmers are less likely to fall victim to central obesity or cancer due to the reason that farmers have routine activities on the farm, which is energy-demanding work that lowers the risk of getting central obesity (48). This can be logically justified as the type of occupation is usually associated with energy expenditure per day. More physical activity brings less fat accumulation on the abdominal wall as lipids or fats are one of the most common energy precursors, especially during the exercise period. Thus, in our study who are regarded as other than a farmer, merchants and government include those participants having no work, are retired, students, and housewives. These study participants may be directly or indirectly favorable for not having labor work or regular exercise. Therefore, early screening of central obesity is vital for all cancer patients to limit its adverse outcomes regardless of their BMI level. It is also supported by a study which reported that centrally obese individuals have poor prognostic outcomes among breast cancer study participants (49).

This study also found that those living in urban areas were less likely to have central obesity by 30% compared to rural residents. This may be due to the reason that accessibility of media, which creates awareness to maintain their weight status, and the probability of getting physician advice is more for urban residents compared to rural residents in Ethiopia (50). Thus, urban residents may undergo regular exercise and modify their diet (which can lower the risk of central obesity and also help to improve the problem). Additionally, cancer patients living in rural areas have less knowledge about the association of central obesity with cancer outcomes. The disparities in cancer risk factors, incidence, mortality, and associated metabolic diseases are also greatly affected by the residency of the patients (51). Therefore, it will be encroached to have health education focused on central obesity for cancer patients.

## Conclusion

This study identifies the magnitude of the hidden type of obesity called central or visceral or abdominal obesity and found that its prevalence was 19.27% among cancer patients attending at oncology unit of the University of Gondar Specialized Comprehensive Hospital. This magnitude was significantly higher among female study participants than that males. Being female, having urban residency, and having occupations other than farmer, merchant, and government were found to be the factors associated with central obesity. Based on our findings, we encouraged screening for early identification of central obesity among cancer patients, and it will be good to manage it, especially for those participants who belong to the associated factors.

## Strengths and limitations of the study

This is the first study that focused on the burden of central obesity and its associated factors in Ethiopia. The study area that fills the knowledge gap on it is taken as the study’s main strength. Being a cross-sectional study design that did not show the cause-effect relationship of the variables, the inability to address the dietary habit of the participants, and the inability to use hip to waist circumference ratio to define central obesity were the study’s limitations. Besides, this study was focused at a single institute that did not generalize the overall cancer patient of Ethiopia, which was another limitation of the study.

## Data availability statement

The original contributions presented in the study are included in the article/supplementary material. Further inquiries can be directed to the corresponding author.

## Ethics statement

The study was conducted in accordance with the ethical principles of the Declaration of Helsinki. Ethical approval was obtained from the University of Gondar Ethical Review Committee. Before running the data collection, written informed consent was obtained from each study participants. All the way through the process of the study, confidentiality was kept for all study participants. The patients/participants provided their written informed consent to participate in this study.

## Author contributions

All authors contributed to the study’s conception and design. All authors performed material preparation, data collection, and analysis. The first draft of the manuscript was written by MM and all authors commented on previous versions of the manuscript. All authors read and approved the final manuscript.
